# Effects of four weeks intermittent hypoxia intervention on glucose homeostasis, insulin sensitivity, GLUT4 translocation, insulin receptor phosphorylation, and Akt activity in skeletal muscle of obese mice with type 2 diabetes

**DOI:** 10.1371/journal.pone.0203551

**Published:** 2018-09-10

**Authors:** Yun Wang, Li Wen, Shi Zhou, Yong Zhang, Xin-Hao Wang, You-Yu He, Allan Davie, Suzanne Broadbent

**Affiliations:** 1 School of Health and Human Sciences, Southern Cross University, Lismore, Australia; 2 Key Laboratory of Exercise Physiology and Sports Medicine, Tianjin University of Sport, Tianjin, China; 3 Department of Health and Exercise Science, Tianjin University of Sport, Tianjin, China; Garvan Institute of Medical Research, AUSTRALIA

## Abstract

**Aims:**

The aims of this study were to determine the effects of four weeks of intermittent exposure to a moderate hypoxia environment (15% oxygen), and compare with the effects of exercise in normoxia or hypoxia, on glucose homeostasis, insulin sensitivity, GLUT4 translocation, insulin receptor phosphorylation, Akt-dependent GSK3 phosphorylation and Akt activity in skeletal muscle of obese mice with type 2 diabetes.

**Methods:**

C57BL/6J mice that developed type 2 diabetes with a high-fat-diet (55% fat) (fasting blood glucose, FBG = 13.9 ± 0.69 (SD) mmol/L) were randomly allocated into diabetic control (DC), rest in hypoxia (DH), exercise in normoxia (DE), and exercise in hypoxia (DHE) groups (n = 7, each), together with a normal-diet (4% fat) control group (NC, FBG = 9.1 ± 1.11 (SD) mmol/L). The exercise groups ran on a treadmill at intensities of 75–90% VO_2_max. The interventions were applied one hour per day, six days per week for four weeks. Venous blood samples were analysed for FBG, insulin (FBI) and insulin sensitivity (QUICKI) pre and post the intervention period. The quadriceps muscle samples were collected 72 hours post the last intervention session for analysis of GLUT4 translocation, insulin receptor phosphorylation, Akt expression and phosphorylated GSK3 fusion protein by western blot. Akt activity was determined by the ratio of the phosphorylated GSK3 fusion protein to the total Akt protein.

**Results:**

The FBG of the DH, DE and DHE groups returned to normal level (FBG = 9.4 ± 1.50, 8.86 ± 0.94 and 9.0 ± 1.13 (SD) mmol/L for DH, DE and DHE respectively, P < 0.05), with improved insulin sensitivity compared to DC (P < 0.05), after the four weeks treatment, while the NC and DC showed no significant changes, as analysed by general linear model with repeated measures. All three interventions resulted in a significant increase of GLUT4 translocation to cell membrane compared to the DC group (P < 0.05). The DE and DH showed a similar level of insulin receptor phosphorylation compared with NC that was significantly lower than the DC (P < 0.05) post intervention. The DH and DHE groups showed a significantly higher Akt activity compared to the DE, DC and NC (P < 0.05) post intervention, as analysed by one-way ANOVA.

**Conclusions:**

This study produced new evidence that intermittent exposure to mild hypoxia (0.15 FiO_2_) for four weeks resulted in normalisation of FBG, improvement in whole body insulin sensitivity, and a significant increase of GLUT4 translocation in the skeletal muscle, that were similar to the effects of exercise intervention during the same time period, in mice with diet-induced type 2 diabetes. However, exercise in hypoxia for four weeks did not have additive effects on these responses. The outcomes of the research may contribute to the development of effective, alternative and complementary interventions for management of hyperglycaemia and type 2 diabetes, particularly for individuals with limitations in participation of physical activity.

## Introduction

It is well known that individuals with type 2 diabetes can gain benefits from regular exercise and weight loss in improving glycaemia control [1]. However, to motivate people participating in regular exercise is a real challenge. In addition, there is a need for suitable types of interventions for those who have limitations of participating in physical activity, such as disability, arthritis or extreme obesity [2, 3]. Therefore, to seek more effective and alternative interventions with better acceptability and minimal side effects for prevention and treatment of diabetes, particularly type 2 diabetes, is still on the agenda of researchers and practitioners.

Intermittent hypoxia intervention (IHI) has been explored as a means of therapy for health conditions in the past decades [4–8]. The term ‘IHI’ used in this article refers to repeated episodes of exposure to hypoxia separated by normoxia conditions. It has been reported that a single bout of one-hour exposure to mild hypoxia, with or without concurrent exercise, had an acute effect on blood glucose and insulin resistance in patients with type 2 diabetes [6, 9]. It has also been demonstrated that a few weeks of exercise in moderate hypoxia resulted in more significant weight loss in individuals with obesity, as compared to exercising with the same or higher intensities under normoxia [10–12]. However, there have been only a small number of reports on the effects of repeated exposure to IHI for several weeks, without a combination of other types of intervention, on fasting blood glucose (FBG) and insulin (FBI) [7, 13], with inconsistent outcomes. For example, Serebrovska et al. [7] reported that in older adults with pre-diabetes, nine sessions of IHI in three weeks with the fraction of oxygen in the inspired air (FiO_2_) of 0.12, did not result in a significant decrease in FBG at the end of the intervention period, but a significant decrease was found one month post the intervention. Schreuder et al. [13] reported that an eight-week exercise-program significantly improved physical fitness of type 2 diabetes patients, but the addition of hypoxia (0.165 FiO_2_) did not potentiate the effects of the exercise on fitness, vascular function and glucose homeostasis. There are also reports from animal trials indicating that a four-week of IHI did not show improvements in weight control, glycaemia control and insulin sensitivity for obese rats, as compared to regular exercise alone or exercise in hypoxia [14, 15]. These discrepancies could be due to differences in research design, such as the level of hypoxia applied, intervention protocols and characteristics of participants. There is a paucity of randomised, controlled clinical trials to determine the effects of IHI on glucose homeostasis in individuals with type 2 diabetes.

The underlying mechanism of hypoxia-induced changes in glycaemia control and insulin sensitivity remains unclear [16]. It has been speculated that in addition to insulin-dependent regulatory pathways, hypoxia may influence the glucose uptake in a similar way as exercise does, i.e. may involve insulin-independent pathways [16]. Insulin action refers to the activation of the insulin signalling cascade stimulated by binding of insulin to its receptor that triggers multiple effects on many biological processes, including glucose and lipid uptake and metabolism, gene expression and protein synthesis, and cell growth, division and survival [17]. Skeletal muscle is the principal site of glucose uptake [18] and one of the main insulin-responsive organs accounting for the regulation of glucose homeostasis under both healthy and diabetes conditions [19]. Approximately 40% of the body mass of human and other mammalian species is made of skeletal muscle [20]. Under hyperinsulinemia, insulin-mediated glucose uptake in skeletal muscle accounts for approximately 75% and 95% of whole body basal glucose disposal at euglycaemia and hyperglycaemia respectively [18]. Specific transporter proteins are required to carry glucose across the cell membranes [21]. It is well known that GLUT1 and GLUT4 are two main transporters of glucose in skeletal muscle [22]. During resting states, GLUT1 expression is low in skeletal muscle with a majority of GLUT4 within intracellular storage vesicles [23]. Glucose can be auto-regulated into muscle cells either by the insulin-dependent process via GLUT4 that is not associated with whole tissue glucose concentration and ATP/ADP ratio; or by the insulin-independent way, which is a high Km hexokinase pathway mediated by the extracellular glucose concentration mainly via GLUT1 [24]. It is widely recognized that the rate of glucose transport in skeletal muscle is a limiting step for glucose uptake at resting conditions, and acute regulation of glucose uptake depends on GLUT4 translocation and expression, for instance, in response to exercise [23]. Studies in both humans and rodents indicate that there are an impaired insulin-stimulated glucose uptake and a reduced rate of glycogen synthesis in insulin-resistant muscles [25, 26]. Decreased glycogen synthesis due to impaired insulin-stimulated glucose transport plays a key role in developing muscle insulin resistance [27]. Thus, skeletal muscle insulin resistance has been considered as the primary defect of type 2 diabetes [25]. The exact mechanism of insulin resistance in skeletal muscle is not fully understood. According to the current literature, the reduced insulin-stimulated glucose uptake resulting from impaired insulin signalling and defects of multiple intracellular cascades including inhibited glucose transport and glucose phosphorylation, and decreased glucose oxidation and glycogen synthesis, plays a key role in the development of insulin resistance in skeletal muscle [28]. Therefore, impaired insulin action in insulin resistant skeletal muscle has been found to be associated with decreased glycogen synthesis due to impaired insulin-stimulated glucose transport [27], and significantly reduced mitochondrial oxidation [25] associated with physical inactivity [29]. It has been well established that exercise intervention can improve insulin action and glycaemic control in individuals with type 2 diabetes [30] that may be the result of an improved oxidative capacity of skeletal muscle [31, 32] that can lead to an improvement in β-cell function [33]. An increased GLUT4 expression in skeletal muscle membrane is an important indicator of exercise-induced improvements [23]. The PI3-kinase-Akt pathway is a known insulin-dependent pathway to stimulate GLUT4 translocation to cell membrane in both humans [34] and rodents [35]. The Akt kinase has been considered as a central node of multiple cell signals [36], and one of the key downstream substrates of the PI3-kinase signalling pathway [37]. The activated Akt is involved in 1) up-regulating GLUT4 translocation and expression in response to insulin stimulation, 2) increasing expression of GLUT1, 3) stimulating glycogen synthesis by suppression of GSK3 to activate glycogen synthase activity, and 4) promoting the rate of glycolysis by up-regulating expression of glycolytic enzymes [36]. It has been suggested that GSK3 may be involved in regulation of glucose homeostasis and the development of insulin resistance [38]. The characteristics of GSK3 include that 1) it has two isoforms, GSK3α and GSK3β, that are widely distributed in mammalian tissues; 2) it is inhibited by its phosphorylation; 3) in response to insulin stimulation, the inhibition of GSK3 is via its phosphorylation by Akt at an N-terminal serine residue (Ser21 in GSK3α and Ser9 in GSK3β); and 4) the inhibition of GSK3 can further dephosphorylate and activate glycogen synthase, contributing to an increase of glycogen synthesis [39, 40]. There has been reports that the both pools of Akt and GSK3β exist in the cytosol, nucleus, and mitochondria [41, 42], and the activity of GSK3β is maintained at a relatively higher level in the mitochondria as compared to that in the cytosol [41]. The translocation of Akt from the cytosol to the mitochondria in response to the stimulation of insulin-like growth factor-1 (IGF-1) can occur within several minutes, and Akt activity in the mitochondria is similar to that in the cytosol [42]. The activity of Akt in mitochondria is rapidly and strongly regulated by intracellular signalling activities and stimulates phosphorylation of GSK3β in mitochondria [42], then leading to potentiating dephosphorylation of pyruvate dehydrogenase (PDH) [43] and enabling its activation to maintain the enhanced mitochondrial oxidative capacity [44]. It has been reported that hypoxia can cause a rise in reactive oxygen species (ROS) generation in skeletal muscle [45] and induce nicotinamide adenine dinucleotide phosphate (NADPH) oxidase activity leading to an increase of Akt activation in myocardial endothelial cells [46]. It has also been demonstrated that NADPH oxidase located in the sarcoplasmic reticulum (SR) is responsible to both an activation of the Ca^2+^ release mechanism and ROS that is not produced by the mitochondria [47]. It is thus speculated that exposure to mild hypoxia may increase NADPH oxidase-dependent Akt activity [46] in the skeletal muscle, causing increased both GLUT4 translocation and mitochondrial oxidative capacity. It is well known that insulin actions at cellular level are via insulin receptor (IR) [17]. Once insulin binds to its receptor, it is auto-phosphorylated. IR auto-phosphorylation involves multiple insulin signalling pathways to catalyse the phosphorylation of intracellular substrates [48, 49]. The IR phosphorylation mainly functions in triggering the phosphorylation of multiple tyrosine residues, and subsequently activation of the receptor kinase and tyrosine phosphorylation of a family of insulin receptor substrates (IRS) proteins (these are commonly referred to as docking proteins) [50]. The phosphorylated IRS are involved in the activation of the PI3-kinase-Akt pathway, which is responsible for most insulin-mediated metabolism [51]. It has been reported that IR phosphorylation is reduced substantially in the skeletal muscle of type 2 diabetes [28]. The effect of IHI on IR phosphorylation and Akt activity has not been examined for diabetes.

Therefore, the aims of this study were, using a randomised, controlled research design, 1) to determine the effects of four weeks IHI on blood glucose homeostasis and insulin sensitivity; 2) to examine the effects of the IHI on the IR phosphorylation, GLUT4 translocation, GSK3 phosphorylation and Akt activity; and 3) to compare the effects of IHI alone with that of exercise in normoxia and in hypoxia. It was hypothesized that 1) exposure to mild IHI (0.15 FiO_2_) for four weeks would result in beneficial effects on glycaemia control and insulin sensitivity; 2) the IHI would cause an increase of GLUT4 translocation and Akt activity; and 3) exercise in hypoxia could induce additive effect as compared to IHI alone or exercise in normoxia, in mice with type 2 diabetes.

We have also measured metabolic substrates including NADH, NAD, lactate, pyruvate, glyceride, glucose and glycogen from the muscle and the liver. The data will be published elsewhere. We focused on the insulin receptor and insulin signalling pathway in this paper.

The observations of cumulative effect of intermittent hypoxia vary from protective value to inducers of pathologic conditions [52]. The positive value of ‘intermittent’ exposure to hypoxia conditions has been realized for training athletes for decades. The recommended altitude range of this model is between altitudes of 1600 m (equivalent to 0.17 FiO_2_) and 3000 m (equivalent to 0.15 FiO_2_); particularly, altitudes > 3000 m (equivalent to < 0.15 FiO_2_) may increase side effects of altitude acclimatization such as mountain illness [53]. Since 1990s, convincing evidence has been shown in the literature that the physiological changes induced by certain hypoxic stimulations not only benefit athletes in training, but also provide a theoretical and practical support for application of hypoxia interventions to reduce risks of certain diseases such as obesity, diabetes and related macro/micro vascular complications [54]. The use of mild hypoxic intervention in our study was with reference to previous reports in the literature [6, 10].

## Materials and methods

### Animal experimental model

Ten-week old male C57BL/6J mice (from Beijing HFK Bioscience Co. Ltd., China) with mean body weight (BW) of 17.5 ± 1.77 (SD) grams (g) were randomly allocated into normal-diet and high-fat-diet groups. They were kept in an air-conditioned room with temperature between 20–25°C, relative humidity 55–65%, and lighting cycle of 12hr on/12hr off, and had free access to water and the respective food. The normal diet contained 14% protein, 72% carbohydrates, and 4% fat [55] and the high-fat diet contained 14% protein, 21% carbohydrates and 55% fat [56]. BW and FBG were measured fortnightly after six hours fasting.

### Study design

According to the literature [57], C57BL/6J mice with FBG of 13.0 mmol/L or above were considered having developed diabetes. After 10 to 12 weeks of feeding, approximately 40% mice in the high-fat-diet group demonstrated FBG levels of 13.0–15.5 mmol/L, with the mean value of 13.9 ± 0.69 (SD) mmol/L, that was similar to the 248.6 ± 8.2 (SE) mg/dL (equivalent to 13.8 ± 0.46 mmol/L) reported by Surwit et al. [57] for diabetic mice. These diabetic mice were randomly allocated into one control (DC) and three intervention groups (n = 7 in each), including the treadmill exercise in normoxia group (DE), resting in hypoxia group (DH), and treadmill exercise in hypoxia group (DHE). All diabetic mice were continuously fed with the high-fat diet during the intervention period. A group of mice (n = 7) was fed with normal diet as the normal control group (NC). The mean FBG of NC was 9.1 ± 1.11 (SD) mmol/L in pre-treatment test. This was in line with the data reported by Surwit et al. [57] that the FBG of normal C57BL/6J mice was around 169.6 ± 6.4 (SE) mg/dL (equivalent to 9.4 ± 0.36 mmol/L).

The number of mice required in this study was justified by an *a priori* estimation, using G*Power3 software [58], to ensure adequate statistical power of 0.8 with type 1 error at 0.05 level and type 2 error at 0.2 level for comparisons using a two-way (treatment, intervention) general linear model with repeated measures (GLMRM). The outcome of the estimation indicated that a minimum of seven mice for each group with a total of 35 mice was required.

**Fig 1:**
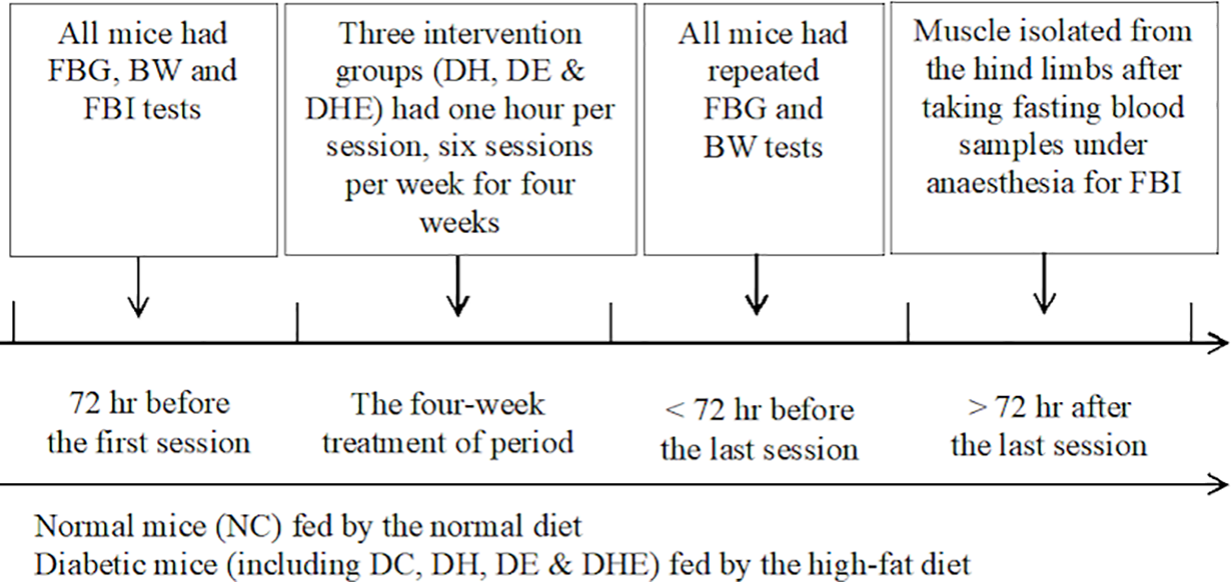
The study design.

The mice were kept in normoxic room air every day, except one-hour exposure to the respective conditions on the intervention days. The NC and DC groups were not exposed to exercise or hypoxia. The three intervention groups were given the corresponding interventions one hour per day, six days per week, for four weeks (Fig 1). Blood samples and BW were obtained pre- and post-treatment period, and muscle specimens were obtained post-treatment period, for analyses of the outcome variables.

### Normoxia and hypoxia environments

The laboratory was located in Tianjin, China, where the average altitude was 3.5 meters, the average barometric pressure was 753.8 mmHg (100.5 kPa), the oxygen partial pressure (PO_2_) was approximately 157.5 mmHg (21.0 kPa) and the FiO_2_ in the air was 0.209 ± 0.001 (SD). The normobaric hypoxia environment was provided by placing the animals in a commercially made facility (Don Whitley H35 hypoxystation workstation and hypoxia tent, U.S.A.), with the FiO_2_ adjusted to 0.15 ± 0.005 (SD) that was equivalent to PO_2_ of approximately 93.0 mmHg (12.4 kPa).

### Exercise program

According to the literature on exercise capacity of obese C57BL/6J mice, the treadmill slope was kept at 0 degree, and velocity was set at 10 cm/s initially, then increased by 2 cm/s every 12 min, that was equivalent to intensities in the range of 75–90% VO_2_max [59].

### FBG and insulin

The blood samples were collected after six hours fasting for measurements of insulin and glucose concentrations, by saphenous venepuncture [60] for pre-treatment measurement, and from inferior vena cava under anaesthesia by sodium pentobarbitone (60 mg/kg) for the post-treatment measurement. The FBG was determined by using a hand-held glucometer (SANNUO, China). The FBI was determined by the ELISA with commercially made Mouse Ultrasensitive Insulin ELISA kits (80-INSMSU-E01, ALPCO).

### Insulin sensitivity

Insulin sensitivity was estimated by the QUICKI, that was calculated as 1/[log(I) + log(G)], where I refers to FBI (μU/mL) and G stands for FBG (mg/dL) [61].

### Skeletal muscle isolation

72 hours post the last intervention session, under anaesthesia (by sodium pentobarbitone, at a dosage of 60 mg/kg body weight), the quadriceps and other skeletal muscles from the hind limbs were collected and stored at -80°C separately.

### Body weight

The BW was measured after six hours fasting, before the four-week intervention. The measurement was repeated within 72 hours of the last intervention session before the blood and muscle sampling (Fig 1).

### Isolation of plasma membrane from quadriceps muscle for testing GLUT4

The membrane of the quadriceps muscle fibres was isolated according to the description by Klip et al. [62] with modification. Briefly, the muscle sample (approximately 100 mg) was rinsed in Solution A (250 mmol/L Sucrose, 50 mmol/L Tris, 0.2 mmol/L EDTA, pH7.4), and centrifuged (MIKRO 120, Hettich America) twice at 120 g for 15 min at 4°C after removing fat, vessels and connective tissues. The mixed supernatant was collected and centrifuged (himac CR22G, HITACHI Japan) at 9000 g for 20 min at 4°C. Then the supernatant was collected and centrifuged (himac CP100WX, HITACHI Japan) at 190,000 g for 60 min at 4°C. The resulting pellet was re-suspended with 2 mL of Solution A and homogenised with a homogeniser in an ice bath. The re-suspended solution was centrifuged (himac CP100WX, HITACHI Japan) at 150,000 g for 16 hr at 4°C in 25% sucrose solution. The 25% sucrose layer was collected, then washed by four-fold dilution in 20 mmol/L Tris-HCl (pH7.4) solution. The mixed solution was centrifuged (himac CP100WX, HITACHI Japan) at 190,000 g for 60 min at 4°C. The resulting pellet was re-suspended with 100 μL of Solution A, homogenised with a homogenizer in an ice bath, and used as a sample (the concentration of protein was determined by the Bradford protein assay kit (Shanghai Institute of Biotechnology (SIB), China). Finally, the sample was boiled in loading buffer for 5 min and stored at -80°C before testing GLUT4 by western blotting [62].

### Preparation of protein extractions from muscle

Frozen muscle sample (40 mg) was ground into fine powder with a mortar and pestle, and then homogenised with a homogeniser in an ice-bath in 2 mL of RIPA lysis buffer (50 mmol/L TrisCl (pH7.4), 150 mmol/L NaCl, 1% Triton X-100, 1% sodium deoxycholate, 1 mmol/L PMSF, 10 μg/mL aprotinin, 0.1% SDS, 2 mmol/L sodium pyrophosphate, 25 mmol/L β-glycerophosphate, 1 mmol/L EDTA, 1 mmol/L Na3VO4, 0.5 μg/mL leupeptin). After ultra-sonicated, the extract was centrifuged (himac CR22G, HITACHI Japan) twice at 12,000 rpm for 20 min at 4°C, and the supernatant was collected and centrifuged (himac CP100WX, HITACHI Japan) at 150,000 g for 60 min at 4°C to remove insoluble materials further. Then the supernatant was collected and the protein concentration was determined by the Bradford protein assay kit (SIB, China). After the supernatant (1 mg protein) was incubated with 2 μg/mL antibody (Rabbit polyclonal anti-IR (Abcam), or Akt Rabbit mAb (CST)) at 4°C for 1 hr, 20 μL protein A/G agarose beads were added, incubated at 4°C overnight, then centrifuged (MIKRO 120, Hettich America) at 3000 rpm for 2 min at 4°C. The pellet was collected and mixed with 2 mL RIPA lysis buffer, then centrifuged (MIKRO 120, Hettich America) at 3000 rpm for 2 min at 4°C, and discarded the supernatant (this procedure was repeated for four more times). The pellet from the last repeat was resuspended with PBS (pH 7.4) (adjusted to approximately 30 μg/μL) and used as a sample for testing phosphorylated IR or Akt by western blotting; or the pellet was resuspended with 1 mL buffer B (25 mmol/L HEPES, 10% glycerol, 1% Triton X-100, 1 mmol/L DTT & 0.1% BSA), centrifuged (MIKRO 120, Hettich America) at 3000 rpm for 2min at 4°C, and the supernatant was discarded (repeated for four more times). Then the pellet was collected and resuspended with 1 mL kinase buffer (50 mmol/L Tris-HCl (pH 7.5), 10 mmol/L MgCl_2_ & 1 mmol/L DTT), centrifuged (MIKRO 120, Hettich America) at 3000 rpm for 2 min at 4°C, and the supernatant was discarded (repeated one more time). The pellet was collected and resuspended with 40 μL kinase buffer (50 mmol/L Tris-HCl (pH 7.5), 10 mmol/L MgCl_2_ & 1 mmol/L DTT). 200 μM ATP and 1 μg GSK3 fusion protein (GRPRTSSFAEG) (CST) were added to the mixture of the above. The mixture was incubated at 30°C for 30 min, and used as a sample for testing phosphorylated GSK3α/β (Ser21/9) by western blotting [63].

### Western blotting for GLUT4, phosphorylated IR, Akt and phosphorylated GSK3α/β (Ser21/9) proteins

The protein extractions were loaded onto 10% SDS-PAGE to separate GLUT4, Akt, phosphorylated GSK3α/β (Ser21/9), respectively; or onto 7.5% SDS-PAGE to separate phosphorylated IR by electrophoresis (SE300-10A-1.0 miniVE complete vertical electrophoresis system, Hoefer). Then the proteins were transferred into polyvinylidene difluoride (PVDF) membranes respectively. Finally, western blotting was performed using anti-GLUT4 (Abcam) (diluted to 1:1000), or Akt Rabbit mAb (CST) (diluted to 1:1000), or Anti-phospho-GSK3α/β (Ser21/9) (CST) (diluted to 1:1000), or Anti-phosphotyrosine-insulin receptor (mouse monoclonal) (Abcam) (diluted to 1:500), as the primary antibody, followed by the horseradish peroxidase (HRP)-conjugated anti-rabbit IgG (Zhongshan Jinqiao Institute of Biotechnology, China) (diluted to 1:10000) for visualizing and quantifying a target protein by the Enhanced Chemiluminescent (ECL, Sigma Co.) and densitometry (the Quantity One Analysis Software (version 4.4.0, BIO-RAD) [62]. GAPDH was used as the reference protein [64].

### Akt activity

Akt immunoprecipitates were collected on protein A/G agarose beads and incubated with GSK3 fusion protein (GRPRTSSFAEG) (CST) at 30°C for 30 min (presented above). GSK3 fusion protein (GRPRTSSFAEG) is a peptide analog of GSK3 and can be phosphorylated by Akt. The measurement of Akt activity was calculated from the phosphorylated GSK3 fusion protein by Akt divided the total Akt in the skeletal muscle [65, 66].

### Statistical analysis

Descriptive results were expressed as means with SD. Two-way GLMRM analysis (IBM SPSS statistics, ver. 22) was used to determine the effect, and interaction between the types of intervention (two control and three intervention groups) and the treatment (pre-post), on the variables measured from the blood. If a significant effect or interaction was detected, then post-hoc tests with Bonferroni adjustment were performed to determine where the differences existed. Statistical significance was set at the alpha level of 0.05.

The measurements of proteins expressions from skeletal muscle were only available at post-treatment. Therefore, one-way ANOVA (IBM SPSS statistics, ver. 22) was used to determine the effects of interventions (five groups) on the expressions of GLUT4, Akt, phosphorylated IR and GSK3 and the Akt activity from skeletal muscle at post-treatment. If a significant effect was detected, then post-hoc test with Bonferroni adjustment was used to determine where the difference existed. Statistical significance was set at the alpha level of 0.05.

This research obtained approval by the Animal Care and Ethics Committee of Southern Cross University (ARA-13/04 and ARA-14/09).

## Results

### FBG

A significant main effect was found for intervention (five groups) (F (4) = 20.45, p < 0.05) and treatment (pre-post) (F (1) = 258.52, p < 0.05). A significant interaction was also detected for intervention by treatment (F (4,1) = 35.74, p < 0.05).

**Fig 2:**
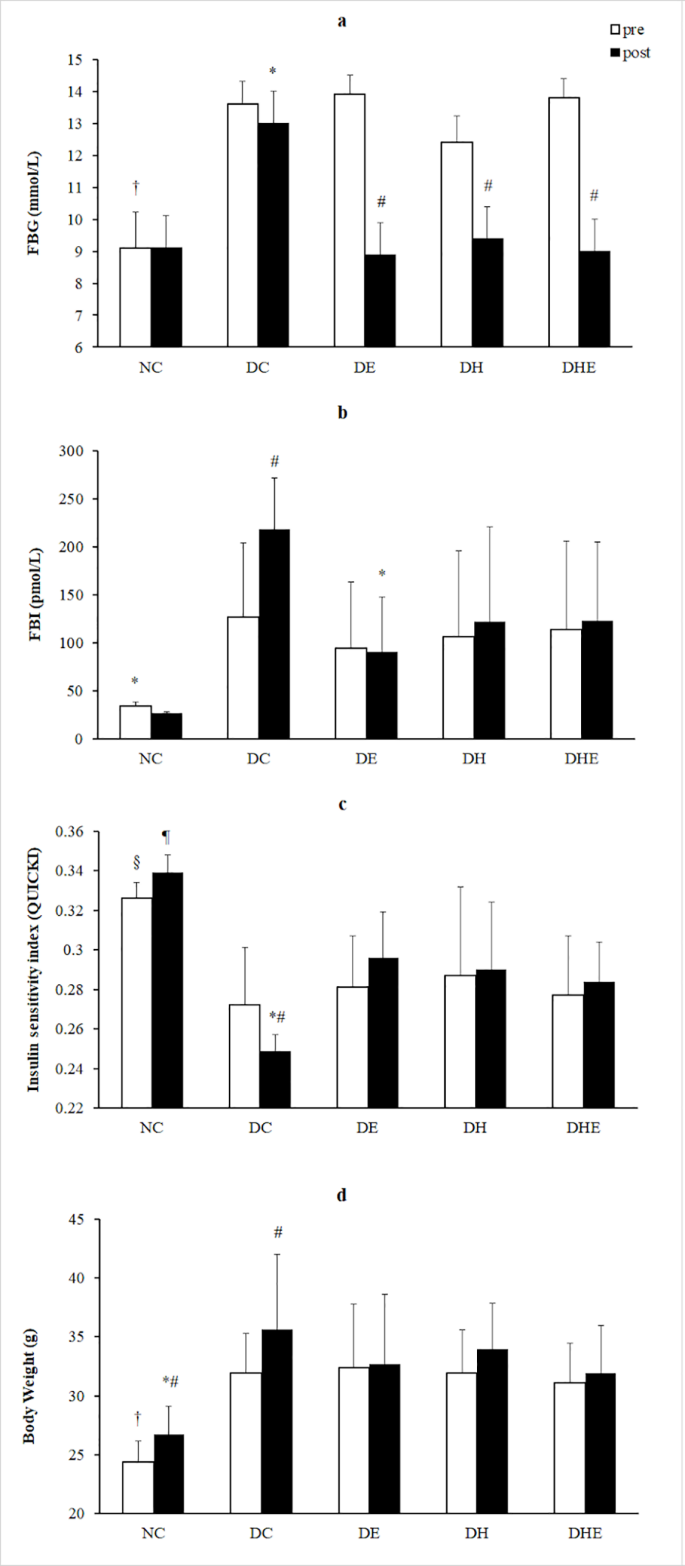
**Effects of four weeks intervention on FBG (a), FBI (b), QUICKI (c) and BW (d).** Data presented are means with SD. †: p < 0.05, NC vs. diabetes group at pre-treatment. §; p < 0.05, NC vs. DC at pre-treatment. ¶: p < 0.05, NC vs. diabetes group at post-treatment. *: p < 0.05, DC vs. other groups at post-treatment. #: p < 0.05, pre vs. post within group.

The post-hoc tests for the intervention by treatment interaction found that the FBG of the diabetes groups was higher than that of the NC group at pre-treatment (F = 50.82, all p < 0.05), but no difference to the NC at post-treatment (p > 0.05) except the DC. The FBG of DC group was significantly higher than all other groups at post-treatment (F = 14.22, all p < 0.05). The FBG of both NC and DC remained unchanged from pre- to post-treatment (both p > 0.05); and that of all three intervention groups dropped significantly from pre- to post- treatment (F = 142.54, 127.53 and 129.84 for DE, DH and DHE respectively, all p < 0.05) (Fig 2A).

### FBI

A significant main effect was found for intervention (F (4) = 4.95, p < 0.05), but not for treatment (F (1) = 3.07, p > 0.05). There was no significant interaction for intervention by treatment (F (4,1) = 2.26, p > 0.05).

The FBI of each intervention group appeared to be higher than that of the NC group at pre-treatment, however this was not statistically significant (all p > 0.05). The FBI of the DC group was significantly increased from pre to post (F = 11.54, p < 0.05), and was higher than that of both the NC and DE groups (both p < 0.05), but not significantly different to the DH and DHE groups at post-treatment (both p > 0.05) (Fig 2B).

### QUICKI

There was a significant main effect of intervention (F (4) = 9.38, p < 0.05), however, no main effect was found for treatment (F (1) = 0.48, p > 0.05). No significant interaction for intervention by treatment was detected (F (4,1) = 2.49, p > 0.05).

The QUICKI of the NC group was higher than that of all diabetic groups at pre-treatment, though a statistical significant difference was found only when comparing to the DC (p < 0.05). The QUICKI of the NC group was remarkably higher than that of all diabetic groups at post-treatment (all p < 0.05), meanwhile, that of the DC group was significantly lower than that of all other groups (p < 0.05). A significantly decreased QUICKI from pre- to post-treatment was only found in the DC (F = 5.60, p < 0.05). All other groups showed a trend of increase in QUICKI from pre- to post-treatment, however no statistically significant difference was detected (F = 1.75, 2.53, 0.10 and 0.47 for NC, DE, DH and DHE respectively, all p > 0.05) (Fig 2C).

### Body weight

A significant main effect was found for intervention (F (4) = 4.75, p < 0.05), and treatment (F (1) = 13.37, p < 0.05). There was no significant interaction for intervention by treatment (F (4,1) = 1.50, p > 0.05).

A significantly increased BW from pre- to post-treatment was found only in the NC and DC control groups (F = 4.18 and 11.03 for NC and DC, both p < 0.05). The BW of all diabetes groups were higher than NC at pre-treatment (all p < 0.05), and differences became non-significant at post-treatment (p > 0.05), except the DC group (p < 0.05) (Fig 2D).

### GLUT4 protein expression in plasma membrane of quadriceps muscle

**Fig 3:**
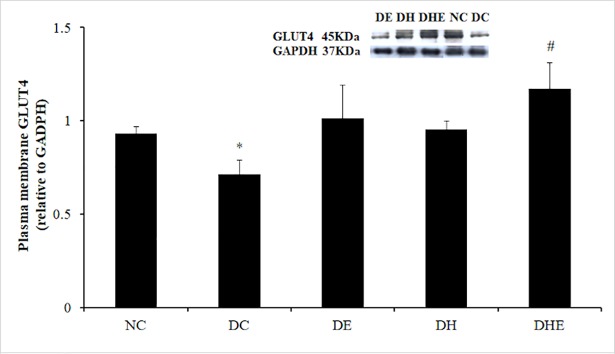
Expression of GLUT4 in plasma membrane of quadriceps muscle at post-treatment. Descriptive data are expressed as mean with SD. *: p < 0.05, DC vs. other groups; #: p < 0.05, DHE vs. all other groups except DE.

There was a significant difference between group means as determined by one-way ANOVA (F (4, 30) = 13.24, p < 0.05). Post-hoc comparisons using the Bonferroni correction indicated that the mean GLUT4 of the DC group (M = 0.71, SD = 0.08) was significantly lower than that of other groups (M = 0.93, 1.01, 0.95 and 1.17, SD = 0.04, 0.18, 0.05 and 0.14, for NC, DE, DH and DHE, respectively) at post-treatment. The mean GLUT4 of the DHE group (M = 1.17, SD = 0.14) was significantly higher than that of other groups (M = 0.93, 0.71 and 0.95, SD = 0.04, 0.08 and 0.05, for NC, DC and DH, respectively) at post-treatment, except DE2 (M = 1.01, SD = 0.18) (Fig 3).

### Phosphorylated IR protein expression

**Fig 4:**
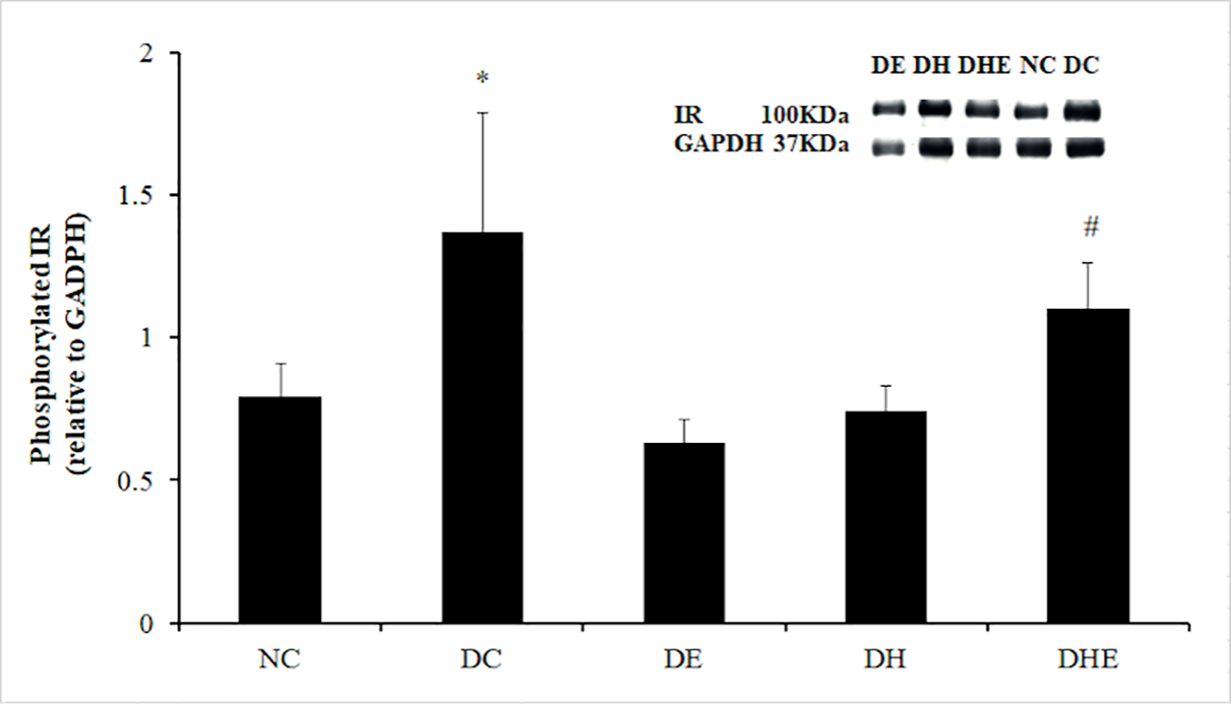
Expression of phosphorylated IR in skeletal muscle at post-treatment. Descriptive data are expressed as mean with SD. *: p < 0.05, DC vs. all other groups except DHE; #: p < 0.05, DHE vs DE and DH.

There was a significant difference between group means as determined by one-way ANOVA (F (4, 30) = 13.89, p < 0.05). Post-hoc comparisons using the Bonferroni correction indicated that the mean phosphorylated IR of the DC group (M = 1.37, SD = 0.42) was significantly higher than that of other groups (M = 0.79, 0.63, 0.74 and 1.10, SD = 0.12, 0.08, 0.09 and 0.16, for NC, DE and DH, respectively) at post-treatment, except DHE (M = 1.10, SD = 0.16). The mean phosphorylated IR of the DHE group (M = 1.10, SD = 0.16) was significantly higher than that of the DE (M = 0.63, SD = 0.08) and DH (M = 0.74, SD = 0.09) at post-treatment. (Fig 4).

### Total Akt protein expression

**Fig 5:**
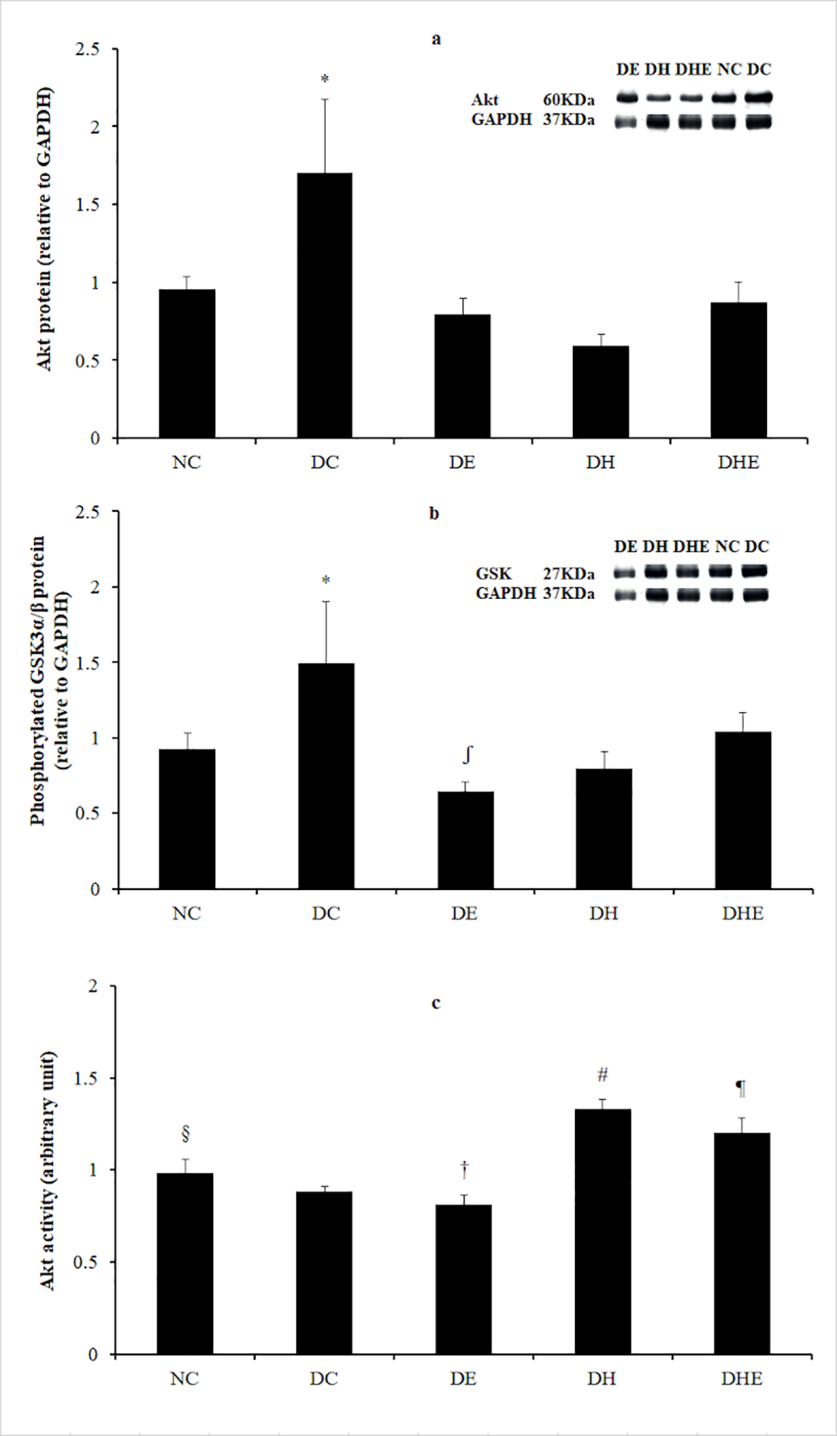
**Expression of Akt protein (a), phosphorylated GSK3α/β protein (b) and Akt activity in skeletal muscle at post-treatment (c).** Descriptive data are expressed as mean with SD. *: P < 0.05, DC vs. all other groups. #: p < 0.05, DH vs. all other groups. ¶: p < 0.05, DHE vs. all other groups. §: p < 0.05, NC vs. all other groups. ʃ: p < 0.05, DE vs. DHE. †: p < 0.05, DE vs. all other groups except DC.

There was a significant difference between group means as determined by one-way ANOVA (F (4, 30) = 23.28, p < 0.05). Post-hoc comparisons using the Bonferroni correction indicated that the mean of total Akt protein in the DC group (M = 1.70, SD = 0.47) was significantly higher than that of all other groups (M = 0.95, 0.79, 0.59 and 0.87, SD = 0.09, 0.11, 0.08 and 0.13, for NC, DH, DE and DHE, respectively) at post-treatment. (Fig 5A).

### Phosphorylated GSK3α/β protein expression

There was a significant difference between group means as determined by one-way ANOVA (F (4, 30) = 16.90, p < 0.05). Post-hoc comparisons using the Bonferroni correction indicated that the mean phosphorylated GSK3α/β of the DC group (M = 1.49, SD = 0.41) was significantly higher than that of all other groups (M = 0.92, 0.64, 0.79 and 1.04, SD = 0.11, 0.07, 0.12 and 0.13, for NC, DH, DE and DHE, respectively) at post-treatment. The mean phosphorylated GSK3α/β of the DE group (M = 0.79, SD = 0.12) was significantly lower than that of DHE (M = 1.04, SD = 0.13) at post-treatment. (Fig 5B).

### Akt activity

There was a significant difference between group means of Akt activity as determined by one-way ANOVA (F (4, 30) = 90.02, p < 0.05). Post-hoc comparisons using the Bonferroni correction indicated that the mean Akt activity of the NC group (M = 0.98, SD = 0.08) was significantly different to that of all other groups (M = 0.88, 0.81, 1.33 and 1.20, SD = 0.03, 0.05, 0.05 and 0.08, for DC, DE, DH and DHE, respectively). The mean Akt activity of the DH group (M = 1.33, SD = 0.05) was significantly higher than that of all other group (M = 0.98, 0.88, 0.81 and 1.20, SD = 0.08, 0.03, 0.05 and 0.08, for NC, DC, DE and DHE, respectively). The mean Akt activity of the DHE group (M = 1.20, SD = 0.08) was also significantly higher than that of other groups (M = 0.98, 0.88 and 0.81, SD = 0.08, 0.03 and 0.05, for NC, DC and DE respectively), except DH (M = 1.33, SD = 0.05). The mean Akt activity of the DE group (M = 0.81, SD = 0.05) was significantly lower than that of all other groups (M = 0.98, 0.81 and 1.20, SD = 0.08, 0.05 and 0.08, for NC, DE and DHE, respectively) at post-treatment, except DC (M = 0.88, SD = 0.03) (Fig 5C).

## Discussion

This was the first randomised, controlled trial on the effects of a long term IHI on FBG, insulin sensitivity and the targeted regulatory factors involved in skeletal muscle glucose uptake and insulin signalling pathway in mice with type 2 diabetes. The major findings were that 1) the four-week IHI (DH) reduced the FBG of the diabetic mice to normal level, that was similar to the effect of exercise in normoxia (DE), however, exercise in hypoxia (DHE) did not induce an additional effect; 2) all three interventions (DE, DH, DHE) resulted in a significant increase of GLUT4 translocation in the skeletal muscle membrane, compared the DC group (Fig 3); and 3) both resting and exercising in hypoxia (DH, DHE) caused a significant increase of Akt activity, whereas exercising in normoxia (DE) did not have this effect (Fig 5C).

### FBG, insulin sensitivity and BW

It has been reported that FBG increases with the progression of insulin resistance in the diet-induced type 2 diabetic C57BL/6J mice [57]. This is underscored by the finding in this study at pre-treatment, as the diabetic mice showed significantly higher FBG and FBI and a reduced insulin sensitivity as compared to the NC group (Fig 2). Continuous high-fat-diet feeding without any intervention resulted in further progress in hyperglycaemia, hyperinsulinaemia, decreased insulin sensitivity, and increased BW in the DC group (Fig 2).

There have been reports that exercise in normoxia or in hypoxia can improve glycaemic control [1, 13–15]. The results of the current study are in line with these reports, while it was the first time to demonstrate that intermittent resting in mild hypoxia for four weeks may also reduce blood glucose to normal level in diabetic mice, similarly to the effects of exercise in normoxia or hypoxia. All diabetes groups showed a higher FBI level compared to NC (non-significant) pre-treatment. It seems the increased insulin level did not reduce the FBG of diabetes mice to the normal range at pre-treatment. At post-treatment the FBI of the DC became significantly higher that the NC and DE while the FBG of all intervention groups were significantly decreased, indicating the beneficial effects of the interventions on insulin sensitivity. This is supported by the significantly higher QUICKI in the three intervention groups than that of the DC (Fig 2).

It has been well established that individuals with type 2 diabetes can benefit from exercise-induced improvements in glycaemia control and insulin sensitivity [30]. It is known that type 2 diabetes results from insulin resistance and islets’ β-cell failure [67], and an improved oxidative capacity of skeletal muscle from exercise may play a key role in improvement of glycaemia control [31]. In return, an improved glycaemic control by regular exercise can lead to an improvement in β-cell function [33] and a further increase of skeletal muscle oxidative capacity [32]. It is an interesting finding that the four-week IHI (DH) resulted in similar changes as the exercise intervention (DE) in the above-mentioned variables, although the underlying mechanism is unclear (will be discussed further below).

In this study, both the NC and DC showed a significant increase of BW pre to post the four weeks (9.4% and 11.6%, respectively), that was possibly due to normal growth in the NC or a combined effect of growth and diet in the DC. Interestingly, the DH group showed a mild weight gain (6.6%, not significant) compared with the control groups, while the two exercise groups (DE 0.9% and DHE 2.6%) had much less change in BW, indicating the effect of caloric expenditure during exercise on BW (Fig 2D).

### GLUT4, IR phosphorylation, GSK phosphorylation and Akt activity

At the cellular level, insulin action via the IR phosphorylation triggers effects on a number of biological processes, including glucose and lipid uptake and metabolism, gene expression and protein synthesis, and cell growth, division and survival [17]. It has been suggested that the process of IR auto-phosphorylation involves multiple insulin signalling pathways to catalyse the phosphorylation of downstream substrates [48, 49], for instance, the PI3-kinase-Akt pathway [51]. It is well known that the activated Akt is involved in increasing GLUT4 translocation and expression in response to insulin stimulation, and stimulating glycogen synthesis by activation of glycogen synthase due to GSK3 phosphorylation [36]. Therefore, it has been considered that GSK3 may be involved in regulation of glucose homeostasis and the development of insulin resistance [38]. For example, enhanced GSK3 activity has been shown in the insulin resistant skeletal muscle of obese Zucker rats [68]. However, there was a report that high-fat feeding did not change GSK3 activity in the skeletal muscle of C57BL/6J mice [69]. The activity of GSK3 is controlled by its phosphorylation [39] and the GSK3 phosphorylation is inversely related to the GSK3 activity [39]. In our study, the IR phosphorylation was higher in the DC group than other groups (non-significant compared to DHE) and the GSK3 phosphorylation was higher (indicating reduced activity) in the skeletal muscle of the DC group compared to the NC group (Fig 5B). This is probably due to the progression of hyperglycaemia, hyperinsulinemia and insulin resistance in the diabetic C57BL/6J mice without any intervention (Fig 2A–2C). Further, the phosphorylation of IR in the DHE group was significantly higher than the DH and DE (Fig 4) and the phosphorylated GSK3 in the DE group was lower than that of both DH (non-significant) and DHE groups, at post-treatment (Fig 5B). That may imply that the four-week exercise in hypoxia had an additive effect on the IR phosphorylation (Fig 4) and GSK3 phosphorylation (Fig 5B) as compared to the four-week exercise or hypoxia alone.

It has been reported that high-fat feeding can result in significantly reduced GLUT4 translocation and insulin-dependent Akt activity in the skeletal muscle of rodents [70]. At the basal state, fasting hyperinsulinemia was observed corresponding to high IR phosphorylation with reduced receptor number and an increase of tyrosine kinase activity in the skeletal muscle of a nutrition-induced diabetes rat model [71]. A reduced GLUT4 translocation has been demonstrated playing a key role in the defect of glucose transport in diabetic skeletal muscle [72]. In this study, the expressions of phosphorylated IR and total Akt proteins at fasting state were significantly higher in the DC than that in other groups, 72 hours post-treatment, while the Akt activity was higher in the NC, DH and DHE than that in the DC (Figs 4 and 5). These would indicate that the hypoxia intervention (alone or with exercise) had an effect on this pathway and that was greater than exercise alone. The GLUT4 translocation can be regulated by the Akt activity, that may explain the current findings (Fig 3). This is in line with the previous reports in the literature [70, 71]. The three interventions can lead to a decreased muscle insulin resistance indicated by a significantly increased GLUT4 translocation (Fig 3), that perhaps contributed to significantly reversed hyperglycaemia and hyperinsulinemia (Fig 2A and 2B) in the three intervention groups.

### Potential mechanisms

It has been suggested that the improved glycaemia control by resting in hypoxia may result from up-regulated whole-body glycolysis pathway, GLUT4 translocation and glucose transport rates [6]; and an acute increase of glucose uptake via insulin-independent way may occur in skeletal muscle under hypoxia exposure [16]. However, there is limited and controversial evidence in the current literature in respect of the suggested up-regulation of glycolysis pathway, particularly in skeletal muscle. First, there has been evidence that O_2_ supply is not limited for cellular respiration until the extracellular PO_2_ drops to a level of less than 5–7 mmHg (0.67–0.93 kPa) [73]. It has been reported that after exposure to sustained hypoxia at 0.10 FiO_2_ for 12 hours, the PO_2_ in the muscle of C57BL6 mice with diet-induced obesity can still be maintained at 13.7 ± 6.0 mmHg (1.83 ± 0.80 kPa) [74]. The oxygen levels in active tissues are tightly regulated by microcirculatory adjustments to ensure oxygen supply [75]. Evidence in the literature showed that diabetic vascular response to 0.08 FiO_2_ was similar to healthy participants [76]. Therefore, it could be extrapolated that one-hour resting at 0.15 FiO_2_ might cause a reduction in skeletal muscle PO_2_ in the DH group, as compared to the NC or DC group, but might not cause significant impairment in O_2_ supply for muscle cellular respiration in the groups of DC, DH and NC. Secondly, reports on direct measurements of cellular biochemical and metabolic processes in skeletal muscle during hypoxia, such as cellular oxygenation, glycolysis and mitochondrial oxidative capacity, are scarce. However, indirect evidence showed that an exposure to 0.15 FiO_2_ did not significantly affect ATP metabolism in healthy rats [77]. Further, it has been demonstrated in the literature that an increased aerobic enzyme activity in skeletal muscle of both animals and humans was stimulated by exposure to hypoxia with atmospheric PO_2_ of 80–100 mmHg (corresponding to approximately 0.06–0.13 FiO_2_). Accordingly, it is speculated that there was a condition of adapted cell hypoxia (maintaining sufficient O_2_ supply and ATP flux) rather than dysoxia (O_2_-limited cytochrome turnover) [69] in skeletal muscle cells of the DH group during acute hypoxia exposure [74, 77]. Thus, the skeletal muscle of the DH group may have an improved mitochondrial oxidative capacity without an increase of anaerobic glycolysis and lactic acid production to drive tricarboxylic acid cycle [77–79]. Finally, it has been indicated that a single bout exposure to hypoxia can result in short term improvement in glycaemia control and the whole-body insulin sensitivity in individuals with type 2 diabetes, and the improvement can be maintained for 24–72 hours [1, 6, 80]. The repeated exposure to moderate hypoxia may have improved oxidative capacity of skeletal muscle in the DH group [77–79], that may play a crucial role in the improvement of glycaemia control, similar to the effect of regular exercise [31].

In this study, the four-week exercise with IHI (DHE) did not show additional effects on glucose homeostasis and insulin sensitivity. As mentioned above, oxygen supply for cellular respiration might be limited if the PO_2_ drops to a level of less than 5–7 mmHg [73]. It has been reported that the skeletal muscle intracellular PO_2_ could be maintained at 23 ± 6 mmHg during resting at 0.10 FiO_2_ for 30 min, whereas it fell to 2–5 mmHg after an exercise at 0.10 FiO_2_ [81] or 3.8 ± 0.3 mmHg during exercise at 0.12 FiO_2_ [82]. It is thus speculated that the skeletal muscle of the DHE group likely developed dysoxia, causing an increased activity in the anaerobic glycolysis pathway that may result in more glucose consumption as compared to the DE group, and more significantly increased glucose consumption as compared to the DH group. All three interventions for four weeks led to normalised FBG with improved insulin sensitivity in diabetic mice, while the effects on basal insulin secretion were different, indicated by slightly reversed fasting hyperinsulinaemia in DE and less increase in DH and DHE (Fig 2B). It could be speculated that resting and exercising at 0.15 FiO_2_ might have different impacts on the insulin regulation system including insulin secretion and clearance, as compared to exercising in normoxia.

Contraction-induced glucose uptake in skeletal muscle has been found mainly depending on the GLUT4 translocation from the intracellular vesicles to cell membrane [23]. An increased GLUT4 translocation has been considered as an important indicator associated with exercise-induced improvement of insulin sensitivity [23]. The evidence from this study demonstrated that the four-week IHI can also cause an increased GLUT4 translocation (expression in the membrane) that would explain the decreased FBG, possibly due to increased glucose uptake. However, the underlying mechanisms of the IHI induced effect on GLUT4 translocation might differ to that of exercise. This speculation is based on that 1) both hypoxia groups (DH and DHE) showed a significant increase of Akt activity and that in the DH group was the highest, whereas regular exercise (DE) did not show this effect; and 2) the DHE had additive effect on the IR phosphorylation as compared to the DH or DE (Figs 4 and 5). The exercise-associated increase of insulin action, indicated by the insulin-dependent PI3-kinase-Akt pathway to enhance expression of GLUT4, has been found in the skeletal muscle of both humans [34] and rodents [35]. Therefore, the exercise training in this study would have caused an up-regulation of the insulin-dependent PI3-kinase-Akt pathway.

The improved insulin sensitivity in response to the hypoxia interventions (DH and DHE) appeared to be via an up-regulation of the insulin-dependent signalling pathway as indicated by a significant increase of GLUT4 translocation in the muscle (Fig 3), accompanied by the lower IR phosphorylation, Akt expression and Akt-dependent GSK3 phosphorylation (Figs 4 and 5), as compared to the DC. Endogenous ROS generation in response to insulin, muscle contraction and hypoxia has been suggested as a second messenger [83] involved in amplified insulin signal transduction [84], the contraction-induced GLUT4 translocation [85], and hypoxia-induced Akt activity [46]. It has been reported that endogenous ROS can improve skeletal muscle insulin sensitivity [86]. Based on the above-evidence [46, 84–86], it is speculated that 1) in response to 0.15 FiO_2_, a lower PO_2_ in the skeletal muscle may cause endogenous ROS generation [47], 2) that could cause an improved skeletal muscle insulin sensitivity [86] and induce NADPH oxidase-dependent Akt activation [46] in the DH group, and 3) the hypoxia-induced Akt activity may have similar function as the contraction-induced GLUT4 translocation in the skeletal muscle of the DE group to increase muscle glucose transport [36, 46, 84] in the muscle of the DH group. This is an insulin-independent way to increase glucose uptake indicated by increased GLUT4 translocation in the DH group. However, with limited resources, we could not determine that the exact mechanisms involved in the insulin-independent way for the improvement of GLUT4 translocation and Akt activity in the skeletal muscle of DH group, and whether there was a cross talk between the insulin-independent and insulin-dependent pathways in the muscle. The higher Akt activity in the two hypoxia intervention groups may also indicate that a long term IHI, alone or with exercise (Fig 5C), can cause additional improvement in mitochondrial oxidative capacity [25, 27, 29, 42–44], compared to exercise in normoxia. Further research is required to address the above-limitations and effects of repeated IHI on mitochondrial function in type 2 diabetes.

## Conclusion

This study produced new evidence that intermittent exposure to mild hypoxia (0.15 FiO_2_) for four weeks resulted in normalisation of FBG, improvement in whole body insulin sensitivity, and a significant increase of GLUT4 translocation in the skeletal muscle, that were similar to the effects of exercise intervention during the same time period, in mice with diet-induced type 2 diabetes. However, exercise in hypoxia for four weeks did not have additive effects on these responses. The outcomes of the research may contribute to the development of effective, alternative and complementary interventions for management of hyperglycaemia and type 2 diabetes, particularly for individuals with limitations in participation in physical activity.

## Supporting information

S1 Appendix The data used in statistical analysis of the study. Body weight and the variables from the blood samples were measured at pre- and post-treatment. The expression of proteins and Akt activity in skeletal muscle were measured at post-treatment. The groups 1, 2, 3, 4 and 5 stand for the groups of NC, DC, DE, DH and DHE, respectively.
(PDF)
