# Supplementary material for: Effects of four weeks intermittent hypoxia intervention on glucose homeostasis, insulin sensitivity, GLUT4 translocation, insulin receptor phosphorylation, and Akt activity in skeletal muscle of obese mice with type 2 diabetes
Source: PLoS One. 2018 Sep 10;13(9):e0203551. doi: 10.1371/journal.pone.0203551 (PMC6130870; doi:10.1371/journal.pone.0203551)
Supplement: S1 Appendix — Body weight and the variables from the blood samples were measured at pre- and post-treatment. The expression of proteins and Akt activity in skeletal muscle were measured at post-treatment. The groups 1, 2, 3, 4 and 5 stand for the groups of NC, DC, DE, DH and DHE, respectively. (PDF) [file pone.0203551.s001.pdf]

Name	Group	Pre weight	Post weight	Pre FBG	Post FBG	Pre insulin	Post insulin	Pre QUICKI	Post QUICKI	IR protein	Akt protein	GSK protein	GLUT4	Akt activity
18L	1	25.0	26.0	9.8	10.2	36.89	28.37	0.319	0.329	1.03	1.08	1.04	0.88	0.96
18R	1	22.0	26.0	8.6	8.5	29.89	28.24	0.335	0.338	0.82	0.96	1.08	0.93	1.13
19L	1	23.0	25.0	7.4	8.0	31.29	25.44	0.340	0.347	0.71	1.02	0.93	0.93	0.91
19R	1	26.0	30.0	10.9	11.3	33.20	27.86	0.319	0.326	0.82	0.83	0.84	0.92	1.01
19O	1	24.0	29.0	9.0	8.4	35.23	27.35	0.325	0.341	0.66	0.85	0.79	0.92	0.93
20L	1	24.0	23.0	8.5	9.4	40.58	26.33	0.321	0.337	0.72	0.91	0.82	0.96	0.90
20O	1	27.0	28.0	9.5	7.7	35.36	23.66	0.323	0.353	0.79	0.97	0.97	1.00	1.00
21L	2	33.0	47.0	14.2	13.4	244.61	254.65	0.243	0.243	1.94	2.39	2.08	0.79	0.87
21R	2	26.0	29.0	13.4	13.0	21.24	192.33	0.330	0.252	1.33	1.58	1.42	0.79	0.90
21O	2	33.0	40.0	13.1	14.1	94.89	260.51	0.273	0.242	1.84	2.22	1.97	0.70	0.89
22R	2	30.0	31.0	13.0	12.0	84.33	229.21	0.277	0.249	0.87	1.11	1.04	0.78	0.94
22O	2	31.0	32.0	13.2	11.2	82.94	147.68	0.277	0.264	1.52	1.82	1.56	0.59	0.86
35R	2	37.0	38.0	14.9	15.0	196.40	156.84	0.248	0.253	0.99	1.27	1.06	0.66	0.83
32R	2	33.0	32.0	13.1	12.5	164.60	285.69	0.256	0.242	1.09	1.50	1.29	0.65	0.86
6L	3	30.0	27.0	14.5	8.8	51.13	43.76	0.290	0.316	0.53	0.63	0.55	0.88	0.87
6R	3	29.0	32.0	13.4	9.6	73.01	79.12	0.281	0.289	0.62	0.69	0.58	1.00	0.84
6O	3	37.0	38.0	13.2	8.1	160.15	180.62	0.256	0.267	0.68	0.83	0.61	0.87	0.73
12L	3	41.0	42.0	13.1	8.6	212.42	48.46	0.249	0.313	0.55	0.72	0.61	1.30	0.85
12R	3	29.0	32.0	14.2	8.3	23.28	146.53	0.323	0.273	0.75	0.91	0.73	0.88	0.80
15R	3	26.0	25.0	14.3	10.6	43.88	29.26	0.297	0.326	0.60	0.81	0.64	0.91	0.79
14L	3	35.0	33.0	14.4	8.0	98.58	110.16	0.268	0.284	0.71	0.92	0.74	1.23	0.80
1L	4	33.0	35.0	14.3	10.9	9.41	20.61	0.370	0.342	0.81	0.69	0.96	0.98	1.39
1O	4	33.0	33.0	13.2	9.6	27.98	29.38	0.318	0.331	0.70	0.57	0.72	0.99	1.26
10R	4	29.0	31.0	13.4	8.1	48.59	137.63	0.295	0.276	0.67	0.50	0.69	0.99	1.38
13L	4	31.0	32.0	14.4	7.7	93.11	125.93	0.270	0.281	0.68	0.51	0.69	0.97	1.35
13R	4	28.0	34.0	13.6	8.5	209.75	317.36	0.248	0.250	0.78	0.62	0.80	0.89	1.29
3L	4	39.0	42.0	14.8	9.4	244.10	99.34	0.242	0.282	0.67	0.56	0.71	0.91	1.27
16O	4	30.0	31.0	15.5	11.8	109.77	126.69	0.263	0.267	0.89	0.71	0.95	0.89	1.34
4R	5	31.0	31.0	14.0	9.0	29.13	77.08	0.314	0.293	1.01	0.77	0.88	1.03	1.14
4T	5	27.0	26.0	14.9	7.5	85.86	71.23	0.272	0.303	1.04	0.88	0.99	1.16	1.13
11L	5	30.0	33.0	14.0	10.4	97.69	127.96	0.270	0.270	0.87	0.65	0.88	1.22	1.35
11R	5	33.0	32.0	13.6	10.3	165.11	56.35	0.255	0.300	1.28	1.03	1.18	0.98	1.15
2L	5	37.0	38.0	13.1	7.7	104.05	210.52	0.270	0.264	1.28	0.98	1.15	1.35	1.17
8L	5	28.0	28.0	13.6	9.3	25.95	58.64	0.320	0.302	1.24	0.94	1.13	1.32	1.20
17R	5	32.0	35.0	13.4	8.9	292.43	262.03	0.240	0.254	1.00	0.86	1.07	1.13	1.24
